# The role of preoperative sildenafil therapy in controlling of postoperative pulmonary hypertension in children with ventricular septal defects

**DOI:** 10.15171/jcvtr.2017.31

**Published:** 2017-09-30

**Authors:** Hamid Bigdelian, Mohsen Sedighi

**Affiliations:** ^1^Department of Cardiovascular Surgery, School of Medicine, Isfahan University of Medical Science, Isfahan, Iran; ^2^Department of Cardiovascular Surgery, Chamran Heart Center, Isfahan University of Medical Science, Isfahan, Iran; ^3^Department of Neuroscience, Faculty of Advanced Technologies in Medicine, Iran University of Medical Science, Tehran, Iran

**Keywords:** Pulmonary Hypertension, Sildenafil Citrate, Cardiac Surgical Procedures, Infant

## Abstract

***Introduction:*** Most of the ventricular septal defects (VSD) are complicated with pulmonary arterial hypertension (PAH) which is the major cause of pulmonary hypertensive crisis and right ventricular failure.

***Methods:*** We reviewed clinical outcomes of 63 infants who underwent cardiac surgery and were divided into three groups. Control group (n=20) did not received sildenafil while group A (n = 22) received drug (0.3 mg/kg) before and after surgery. Group B (n=21) received drug at the initiation of surgery. Demographic data, preoperative and postoperative variables were compared among the patients.

***Results:*** Patients in the group A had lower preoperative pulmonary arterial pressure (PAP) compared to other groups (*P *< 0.001). Also, patients in control group had longer cardiopulmonary bypass time (*P *< 0.05). Postoperative PAP in patients of group A and B decreased significantly compared to control group (*P *< 0.001). Also, pre- and postoperative PVR (pulmonary vascular resistance) showed a significant decrease in group A compared with control and group B (*P *< 0.001). The intubation time in patients of the control group was significantly more prolonged compared with patients of group A and B (P < 0.001). Moreover, the length of ICU stay was significantly longer in patients of control group compared with group A and B (*P *< 0.001).

***Conclusion:*** Preoperative sildenafil therapy seems to be effective and safe to prevent postoperative PAH and pulmonary hypertensive crisis in children with ventricular septal defects and has a positive impact on postoperative care.

## Introduction

The most common congenital heart disease (CHD) is the ventricular septal defects (VSDs) with the incidence of 40%. Most of the VSDs are complicated by pulmonary arterial hypertension (PAH) due to high pulmonary blood flow.^1^ PAH associated with VSDs in children, defined by an increase in pulmonary arterial pressure (PAP), is a major cause of the pulmonary hypertensive crisis and right ventricular failure (RVF).^2,3^ There are many treatment choices for PAH including prostacyclin infusion and oral calcium channel blockers and vasodilators. Oral sildenafil, a phosphodiesterase inhibitor (PDEI), is confirmed to selectively reduce pulmonary vascular resistance (PVR) in various types of PAH in children. Moreover, sildenafil is well tolerated and available as an oral preparation which is its advantage in children with PAH.^4,5^ This study was aimed to investigate the effect of oral sildenafil as monotherapy in controlling pre and postoperative PAH in children with large VSD undergoing cardiac surgery.

## Methods

### Study population

This randomized controlled study was performed between 2012 and 2014 on 63 infants with large VSD and moderate to severe PAH (pulmonary arterial to aortic [PA/AO] pressure ratio >0.7) who underwent surgery. All of infants underwent right heart catheterization (RHC) in the two months before surgery. Aortic pressure, PA pressures and oxygen saturations were measured directly during RHC. Preoperative and intraoperative transthoracic echocardiography (TTE) was done by a pediatric cardiologist to confirm the medical diagnosis. Preoperative and postoperative PA/AO, PVR and systemic vascular resistance (SVR) were calculated. The patients were randomly assigned to three groups. The control group included 20 patients who did not receive sildenafil. Group A include 22 patients who received sildenafil (0.3 mg/kg every 4 hours) through the nasogastric tube (NGT) or orally one week before surgery and continued 24–48 hours after surgery. Group B consisted of 21 patients who received sildenafil only at the initiation of cardiopulmonary bypass (CPB) and continued with the same protocol. AOP and PAP were measured via the intraoperatively placed PA and arter monitoring line during 48 hours of ICU stay. Infants with persistent PAP received additional vasodilators such as intravenous nitroglycerin. PVR and SVR were calculated from standard equations. Demographic data, cardiac diagnoses, preoperative and postoperative PAP and AOP, and other variables of all patients in each group were compared between the three groups.

### Statistical analysis

Data were analyzed using the Statistical Package for the Social Sciences 22 (SPSS Inc., Chicago IL, USA) statistical software package. Continuous values were expressed as mean ± standard deviation and analyzed using ANOVA. Pearson chi-square was used to examine the categorical data. *P* value less than 0.05 were considered significant.

## Results

### Demographics data

The study population consisted of 39 males (62%) and 24 females (38%). Among the patients with VSD, 60 infants had permembranous VSD and 3 infants had muscular VSD. Concomitant anomalies included VSD +PDA (patent ductus arteriosus) and VSD+ASD (atrial septal defect). Demographic data, types of VSD and concomitant anomalies are shown in Table 1.

**Table 1:** Demographic and baseline characteristics of patients

**Variables**	**(n = 63)**	**Percent**
Gender		
Male	39	62
Female	24	38
VSD type		
Per membranous	60	95
Muscular	3	5
Diagnosis defect		
VSD	10	15
VSD+PDA	47	75
VSD+ASD	6	10

VSD: ventricular septal defect; PDA: patent ductus arteriosu; ASD: atrial septal defect.

### Preoperative data

**Figure 1:**
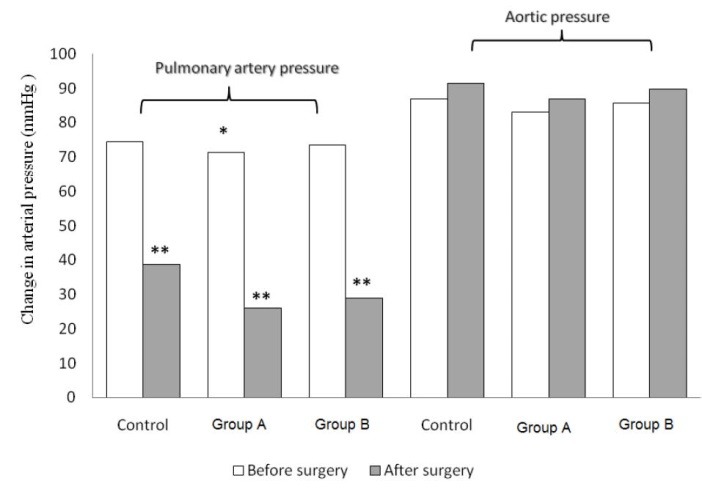
Comparison of pulmonary arterial pressure and aortic pressure before and after surgery among the experimental groups.

The mean age, weight and body surface area (BSA) of patients in each group were illustrated in Table 2. As shown in Table 2, mPAP was significantly lower in group A that received preoperative sildenafil compared with control and group B (71.3 ± 2.9 versus 75.4 ± 3.0 and 73.3 ± 3.2 mm Hg, *P* = 0.001) (Figure 1). Likewise, preoperative PVR has been shown a significant decrease in group A compared with control and group B (1088.5 ± 53.3 versus 1161.8 ± 53.2 and 1125.1 ± 57.1, *P* = 0.001). There were no statistically significant differences among the three groups regarding preoperative variables including aortic pressure, PA/AO pressure ratio, cardiac index (CI), SVR and O2 saturation.

**Table 2:** Comparison of preoperative variables among the control group and group A and B

**Variables** ^a^	**Control group (n = 20)**	**Group A (n = 22)**	**Group B (n = 21)**	***P*** ** value**
Age (month)	5.4±0.6	5.4±0.5	5.7±0.3	0.123
Weight (kg)	6.4±0.6	6.6±0.6	6.8±0.5	0.206
BSA(m^2^)	0.3±0.1	0.3±0.07	0.3±0.1	0.433
CI (L/min/m^2^)	4.1±1.1	3.7±0.9	3.6±1.1	0.381
mPAP (mm Hg)	75.4±3.0	71.3±2.9	73.3±3.2	0.001*
mAOP (mm Hg)	86.7±6.2	83±7.6	85.5±8.2	0.254
PA/AO pressure ratio	0.86±0.6	0.85±0.6	0.85±0.7	0.862
PVR (dynes-sec-cm^-5^)	1161.8±53.2	1088.5±53.3	1125.1±57.1	0.001*
SVR (dynes-sec-cm^-5^)	4362.4±334	4135.4±414	4294.3±440	0.175
Oxygen saturation (%)	86.9±1.1	86.6±1.1	87±2.04	0.764

BSA: Body Surface Area, CI: Cardiac Index, PVR: Pulmonary Vascular Resistance, SVR: Systemic Vascular Resistance, mPAP: mean Pulmonary Arterial Pressure; mAOP: Mean Aortic Pressure; PA/AO: Pulmonary Arterial to Aortic. ^a^All parameters are presented as mean ± standard deviation and analyzed using ANOVA.

### Postoperative data

There was no significant difference in aortic cross clamp (ACC) time, but patients in control group had a longer cardiopulmonary bypass (CPB) time (105.6 ± 18.5 versus 93.1 ± 13.3 and 97.1 ± 16.7 min, *P* = 0.049). Postoperative mPAP decrease after surgery in three groups, but was significant in group A and group B that received sildenafil (25.98 ± 0.86 and 28.84±0.38 versus 38.6 ± 0.9 mm Hg, *P* = 0.001) (Figure 1). Consequently, the PA/AO pressure ratio had a significant decrease in patients of group A and B compared with control group (0.29 ± 0.02 and 0.32 ± 0.03 versus 0.42 ± 0.03, *P* = 0.001). Likewise, PVR in group A decreased significantly compared with control and group B (282.9 ± 15.4 vs 508.6 ± 16.8 and 334.1 ± 6.8 dynes-sec-cm-^5^, *P* = 0.001). Intubation time and length of ICU stay was significantly more prolonged in patients of control group compared with group A and B (*P* = 0.001) (Table 3). There were no mortality events in either group. Three patients in control group who did not get sildenafil experienced pulmonary hypertensive crisis (15%). There were not seen any sign of sternal infection, endocarditis or bleeding.

**Table 3:** Comparison of postoperative variables among the control group and group A and B

**Variables** ^a^	**Control group (n=20)**	**Group A (n=22)**	**Group B ( n=21)**	**P value**
ACC time (min)	61.9±8.9	66.8±11.1	68.1±12.3	0.169
CPB time (min)	105.6±18.5	93.1±13.3	97.1±16.7	0.049*
mPAP (mm Hg)	38.6±0.9	25.98±0.86	28.84±0.38	0.001*
mAOP (mm Hg)	91.4±7.9	86.90±7.6	89.71±8.2	0.184
PA/AO pressure ratio	0.42±0.03	0.29±0.02	0.32±0.03	0.001*
Oxygen saturation (%)	95.7±0.73	96.2±0.75	96.1±1.03	0.094
PVR (dynes-sec-cm^-5^)	508.6±16.8	282.9±15.4	334.1±6.8	0.001*
SVR (dynes-sec-cm^-5^)	4553.2±298	4363.2±399	4505±421	0.242
Intubation time (hours)	12.3±1.0	8.4±0.9	9.07±1.3	0.001*
ICU stay (hours)	75.3±3.0	70.09±4.7	70.71±2.0	0.001*

ACC: aortic cross clamp; CPB: cardiopulmonary bypass; mPAP: mean pulmonary arterial pressure; mAOP: mean Aortic pressure; PA/AO: pulmonary arterial to aortic; PVR: pulmonary vascular resistance, SVR: systemic vascular resistance; ICU: intensive care unit. ^a^All parameters are presented as mean ± standard deviation and analyzed using ANOVA.

## Discussion

In this study, we investigated the effects of sildenafil therapy before and after CHD in infants. Our finding has demonstrated that use of oral sildenafil was successful in declining PAP and eliminating the risk of pulmonary hypertensive crisis after surgical VSD repair. Our results are parallel with the studies conducted by El Midany et al^4^ and Peiravani et al^5^ reported the efficacy of sildenafil to control postoperative PAH and preventing the crisis in infants after congenital heart surgery. Also, our results support previous trials which have been shown evidence of PAH treatment in VSD patients by preoperative use of sildenafil.^6-9^

PAH secondary to VSD remains a main cause of postoperative morbidity and mortality. Large VSD and large left-to-right shunt result in increase of PVR that lead to respiratory symptoms and poor growth in children with VSD. Hence, closure of a large VSD is preferred to do during early stage of life before increase of PVR. For patients who undergo surgical repair of VSD, long-term outcomes are hopeful with normal growth and good quality of life after more than 10 years.^10,11^ During the last years, there has been a remarkable progress in the management of PAH to decline its severity and manage both preoperative and postoperative PAH in order to improve surgical outcomes. Postoperative residual PAH commonly increase the risk of pulmonary hypertensive crisis and right ventricular dysfunction (RVD).^12^ From clinical point of view, residual PAH after surgical closure of large VSD and left-to-right shunt may lead to adverse hemodynamic instability. The sensitivity of right ventricle (RV) to afterload changes is more than left ventricle (LV). Hence, RVD in patients with severe residual PAH is another complication which is aggravated by prolonged CPB time and increased PAH. Thus, the role of PAP management is crucial to minimize postoperative morbidity and Mortality.^13-15^

Sildenafil mainly act on smooth muscles of pulmonary vessels and works synergistically with inhaled nitric oxide (NO).^16,17^ The efficacy of sildenafil in treatment of PAH with minimal systemic vasodilatation lead to increasing use of it in pediatric patients. Furthermore, adequate oral absorption with minimal side effects and rapid onset of action make it treatment of choice for management of PAH.^18^

### Study limitations

There were several limitations in this study such as relatively small sample size and absence of careful long-term follow-up. Long-term studies are required to determine long-term effects of sildenafil and its side effect on infants who receive this drug.

## Conclusion

Preoperative sildenafil therapy seems to be effective for management of PAH, especially secondary to large VSD and large left-to-right shunt. Also, preoperative sildenafil therapy has favorable effects on postoperative care and controlling PAP, which result in less intubation time and ICU stay. Nevertheless, further investigations are necessary to obtain the efficacy, optimal dosing in pediatrics and the safest route of use in children who undergo surgical VSD repair.

## Ethical approval

All patients gave written informed consents and the study was approved by our local Ethics Committee.

## Competing interests

The authors declare there is no conflict of interest.

## Acknowledgments

The authors would like to thank all the patients and all the individuals who gave us the chance to carry out this study. They are also grateful for support and participation of their colleagues and the nurses of the intensive care units of Chamran Heart Center.
